# From Inception to Implementation: Strategies for Setting Up Pulmonary Telerehabilitation

**DOI:** 10.3389/fresc.2022.830115

**Published:** 2022-05-02

**Authors:** Catarina Duarte Santos, Fátima Rodrigues, Cátia Caneiras, Cristina Bárbara

**Affiliations:** ^1^Instituto de Saúde Ambiental (ISAMB), Faculdade de Medicina, Universidade de Lisboa, Lisboa, Portugal; ^2^Unidade de Reabilitação Respiratória do Hospital Pulido Valente, Centro Hospitalar Universitário Lisboa Norte, Lisboa, Portugal; ^3^Laboratório de Microbiologia na Saúde Ambiental (EnviHealthMicroLab), Instituto de Saúde Ambiental (ISAMB), Faculdade de Medicina, Universidade de Lisboa, Lisboa, Portugal; ^4^Instituto de Medicina Preventiva e Saúde Pública, Faculdade de Medicina, Universidade de Lisboa, Lisboa, Portugal; ^5^Healthcare Department, Nippon Gases Portugal, Vila Franca de Xira, Portugal; ^6^Serviço de Pneumologia, Centro Hospitalar Universitário Lisboa Norte, Lisboa, Portugal

**Keywords:** telerehabilitation implementation, exercise, self-management, face-to-face, home-based

## Abstract

**Background:**

The emergence of innovative technology-enabled models of care is an opportunity to support more efficient ways of organizing and delivering healthcare services and improve the patient experience. Pulmonary telerehabilitation started as a promising area of research and became a strategic pandemic response to patients' decreased accessibility to rehabilitation care. Still, in the pre-COVID-19 era, we conducted a participatory study aiming to develop strategies for setting up pulmonary telerehabilitation as a person-centered digitally-enabled model of care.

**Methods:**

We performed operational participatory research between June 2019 and March 2020 with the engagement of all stakeholders involved in the implementation of pulmonary telerehabilitation, including 14 people with Chronic Obstructive Pulmonary Disease. Patients were assessed subjectively and objectively pre and post a 3-month pulmonary rehabilitation program including exercise and education, which started in a face-to-face hospital setting during the first month and continued as a home-based, remotely supervised exercise training intervention.

**Results:**

Five major groups of requirements targeted operational strategies for setting up pulmonary telerehabilitation: (1) pulmonary rehabilitation core principles, (2) quality and security standards, (3) technological functionality, (4) home environment appropriateness, and (5) telesetting skills. There was a statistical significance in the median change in the CAT score from 15.5 to 10.5 (*p* = 0.004) and in the PRAISE score from 49.5 to 53.0 (*p* = 0.006). Patients' mean levels of satisfaction regarding rehabilitation goals achievements were 88.1 ± 8.6% and the mean levels of satisfaction regarding the telerehabilitation experienced as a model of care were 95.4% ± 6.3%.

**Conclusions:**

The success of telerehabilitation implementation was grounded on stakeholder engagement and targeted strategies for specific setup requirements, achieving patients' high satisfaction levels. Such operational experiences should be integrated into the redesigning of upgraded telerehabilitation programs as part of the solution to improve the effectiveness, accessibility, and resilience of health systems worldwide.

## Introduction

Over the last decade, the progressive adoption of technology in healthcare has brought about a significant revolution in health service delivery and in the interactions between patients and healthcare providers (1). Pulmonary rehabilitation, as a comprehensive patient-tailored intervention including exercise training, education, and behavior change, faces the ultimate challenge of succeeding in improving patients' long-term adherence to health-enhancing behaviors (2). To optimize such a purpose, the strategy of providing a real-life setting by means of home-based programs with remote supervision comes as a realistic rationale, as it might increase both patient engagement and pulmonary rehabilitation responsiveness with enduring effectiveness in everyday life. As an add-on, the emergence of innovative technology-enabled models of care presents new and exciting opportunities (3) where telerehabilitation provides delivery of pulmonary rehabilitation programs either by means of a telephone, a website or mobile application, or via video-conferencing (4). With established scientific evidence (5–9), telerehabilitation in chronic respiratory disease presents similar outcomes compared to traditional in-person, center-based pulmonary rehabilitation (10).

While acknowledging that digital solutions enable more efficient ways of organizing and delivering healthcare services, it is of great importance that the design of such models of care meets the needs of people and health systems. Participatory research aims for the convergence of the perspectives of science and practice (11), empowering co-researchers to rethink established programs, services, and policies and enabling the co-creation of products the community can utilize (12). Beyond inception, innovative solutions through new technologies must be thoughtfully implemented to suit the local context, taking into account not only the best clinical practice but also organizational changes and, very importantly, improved patient experience. To succeed in such a purpose, the active engagement of all parties is essential, including patient involvement. Unaware of a challenging pandemic scenario to come, we conducted participatory research including patients within stakeholders, with the aim to develop strategies for setting up pulmonary telerehabilitation as a person-centered digitally-enabled model of care.

## Materials and Methods

Operational participatory research with the engagement of all stakeholders involved in the implementation of pulmonary telerehabilitation is described in Table 1.

**Table 1:** Participatory research framework.

**Participatory research framework**		
**Stakeholders**	**Interactions**	**Operational participation**
Patients	Assessment sessions (2x) Exercise sessions (24x) Educational sessions (6x) Domiciliary visit (2x) Telerehabilitation (12x)	Research design on protocol sequence of assessments Taking part in decisions about the content of exercise and educational sessions Collaboration on tutorial for telerehabilitation Onset of domiciliary conditions for telerehabilitation Design of telerehabilitation sequence of communication
Caregivers	Domiciliary visit (2x) Telerehabilitation (12x)	Onset of domiciliary conditions for telerehabilitation
Rehabilitation team	Educational sessions (6x)	Setting up educational sessions from the Living-Well with COPD Self-management program customized to group intervention Decision to provide additional care in case of benefit of an individual approach
Secretariat staff	Daily backup operations	Scheduling recruitment Administrative records Overall service process outcomes
Technology and communication staff	Installing (1x)	Making decisions on space organization and distribution of net points and computers within hospital facilities
Logistics and transportation staff	Domiciliary visit (monthly)	Making decisions on the best conditions to provide transport and delivery of material and equipment at the patient's home
Department administrator	Periodic meetings	Assuring logistic conditions for implementation within hospital facilities Scheduling support from logistics and transportation staff to support domiciliary visits with a physiotherapist
Service director	Periodic meetings	Scheduling timeline of implementation Monitoring project execution
Hospital administration	Communication reports	Approval of institutional proceedings

Patient engagement included people with Chronic Obstructive Pulmonary Disease (COPD) referred for pulmonary rehabilitation at Hospital Pulido Valente in Lisbon, Portugal (June 2019 to March 2020). Participants had a confirmed COPD spirometry diagnosis and were stable without any clinical exacerbation or hospitalization in the previous 6 weeks. Exclusion criteria were uncertainty to commit with program adherence for 3 months; exercise-compromised clinical conditions (metastatic neoplasia, infectious or unstable cardiac diseases, osteoarticular, neuromuscular, unstable psychiatric or cognitive disorders); referenced candidate to lung transplant; multi-resistant bacterial infection/colonization; and any formal exercise contra-indications. Given the extenuating circumstance of COVID-19, which was declared a pandemic on 11 March 2020 by the World Health Organization, as defined by the CONSERVE 2021 Statement (13), ongoing research activity was affected. An important trial environment factor that was affected was the feasibility, as non-urgent clinical activity was suspended by the Board of the Hospital in line with national instructions by the Portuguese Directorate-general for Health. Suspended recruitment and exclusion of outcomes objective face-to-face evaluations were additional factors that directly impacted the trial. Mitigation strategies applied were the completion of the intervention phase for subjects already enrolled, and data collection was conditioned with remote subjective outcomes assessment. Despite the initial study design of patients randomly allocated either for a traditional face-to-face ambulatory pulmonary rehabilitation program (control group) or to a pulmonary rehabilitation program with telerehabilitation, given the described research limitations per-protocol suspension, this article presents a proof of concept with a randomized telerehabilitation group, leaving out the control group. The research was performed in accordance with the Declaration of Helsinki, with informed consent given prior to any proceeding. Ethical approval was obtained from the Ethics Committee of Centro Hospitalar Universitário Lisboa Norte, and Centro Académico de Medicina de Lisboa (number 43/17).

Patient clinical objective assessment was performed in accordance with the relevant guidelines and included: exercise capacity with a cardiopulmonary exercise test (14–16); functional capacity with the 6-min walk test (17–19) and the 1-min sit-to-stand test (20–22); daily activity functional capacity with the Glittre test (23–26) and a handgrip strength test (27–29). The subjective assessment included health-related quality of life by means of the EuroQoL 5 dimension Visual Analog Scale (30, 31) and the COPD Assessment Test (CAT) (32, 33); dyspnea with the modified Medical Research Council dyspnea scale (mMRC) (34) and the London Chest Activity of Daily Living scale (LCADL) (35, 36); anxiety and depression using the Hospital Anxiety and Depression Scale (HADS) (37); self-efficacy by means of the Pulmonary Rehabilitation Adapted Index of Self-efficacy Scale (PRAISE) (38, 39); and cognitive function by application of the Montreal Cognitive Assessment test (MoCA) (40, 41).

**Figure 1:**
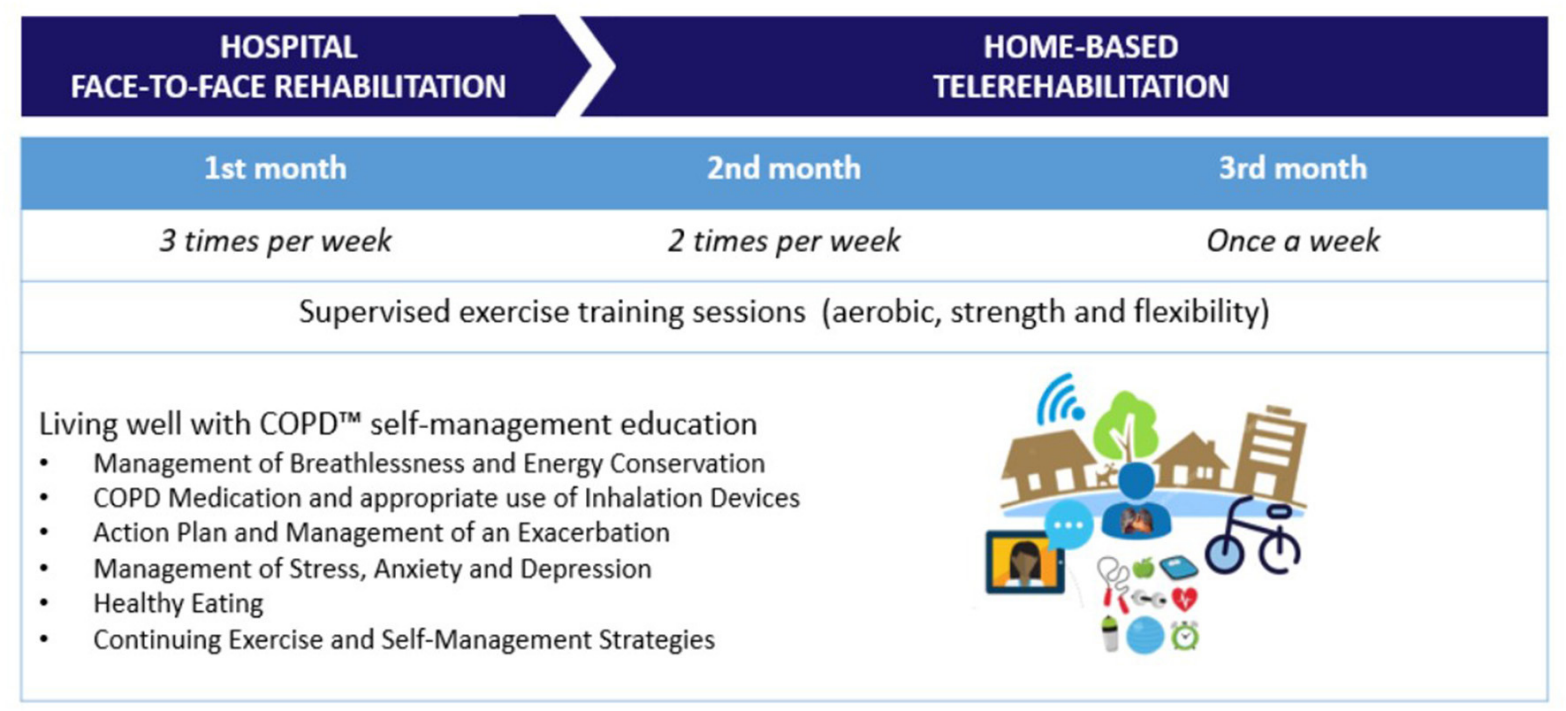
From hospital face-to-face rehabilitation to home-based telerehabilitation.

Pulmonary rehabilitation was designed as a 3-month group intervention with a multidisciplinary program (respiratory physician, physiotherapist, dietitian, and psychologist), including 6 self-management education sessions based on the Living-Well with COPD™ program (42) and 24 exercise training sessions supervised by the physiotherapist, as presented on Figure 1. The exercise training intensity was based on cardio-pulmonary exercise testing with an incremental protocol using a bicycle ergometer. Aerobic exercise training included modalities with a bicycle ergometer, treadmill, upper-limb ergometer, and rowing machine in the hospital face-to-face rehabilitation and bicycle and upper-limb ergometers domiciliary allocated for telerehabilitation. Aerobic exercise duration was 30–45 min, with targeted training intensities between 60 and 80% VO_2_peak (43), initiated at the maximum tolerated intensity for a round of 10 continuous minutes had been accomplished with SpO_2_ > 90% and a Modified Borg Scale score below 5 either for dyspnea or muscle fatigue. Progression was executed each session, increasing first the duration and then the intensity, according to patient tolerance. Strength exercise training included the use of weighted balls, dumbbells, and elastic bands for three exercises with three sets of 10 repetitions with breathing control and oximetry monitoring during pauses between sets to monitor SpO_2_ > 90%. Flexibility training included four global muscle chain exercises on stall bars (posterior, anterior, and lateral) and specific stretching exercises according to the selected ergometers during the aerobic training (quadriceps, hamstrings, calf, biceps, triceps, pectoralis, and rhomboids). Also, at the end of each exercise session, there was a 10-min routine of laying down relaxation with dimmed light, soothing music, and guided breathing control.

Patients were enrolled in the program in a hospital setting during the first month and were upgraded to a home-based real-time monitoring video-call telerehabilitation after 3 months. The VSee platform (VSee, California, United States of America) was used for telerehabilitation, as it fulfilled the requirements of the General Data Protection Regulation and the Health Insurance Portability and Accountability Act; additionally, this was the official selection by the NASA Space Station to provide secure video conferencing. Baseline logistics for telerehabilitation were a hospital computer exclusive to telerehabilitation, including a webcam and earphones. Also, to enable telerehabilitation implementation without excluding patients that could not afford the expenses of the equipment, technology, and communication for remote care, each patient was provided for domiciliary usage a stationary bicycle (Domyos Essential 2 Bike, DECATHLON, China), an upper-limb cycle ergometer (YF612 minibike, Tecnovita—BH, Portugal), a digital oximeter (PRIM oximeter Md300C15D, PRIM, Spain), a blood pressure and pulse rate monitor (OMRON M2, OMRON Healthcare Co. Ltd., Japan), an android 4G tablet (Vodafone Smart TAB N8, Vodafone, China) including earphones and internet card, and a Borg scale. This represented an initial investment of 570€ per telerehabilitation kit, delivered and collected from each patient's home and continuously reallocated to new patients. Additionally, there were operating costs with data communication of 40€ per patient within the program aside from the hospital support provided by human resources and services.

When the pulmonary rehabilitation program concluded, patients used a scale from 0 to 100% to report their level of satisfaction with the rehabilitation goals achievement and their level of satisfaction with pulmonary rehabilitation with telerehabilitation experienced as a model of care.

Statistical analysis and data management were performed using the Statistical Package for the Social Sciences (SPSS) version 25.0 (SPSS Inc., Chicago, IL, USA). Descriptive statistics, such as frequencies, were presented as percentages, and data were expressed as median and interquartile ranges. Changes in pulmonary rehabilitation outcomes were analyzed by related-sample Wilcoxon Signed Rank test with a *p*-value of less than 0.05 being considered statistically significant.

## Results

Table 2 presents baseline patient characteristics, including subjective and objective assessments.

**Table 2:** Baseline patient characteristics.

	**Total (*N* = 14)**
Male, *n* (%) Age (years; median ± IQR)	10 (71.4) 65.0 ± 17
**Subjective assessment** EuroQoL—VAS (%; median ± IQR) CAT (score; median ± IQR) mMRC (score; median ± IQR) LCADL (score; median ± IQR) HADS A (score; median ± IQR) HADS D (score; median ± IQR) MoCA (score; median ± IQR) PRAISE (score; median ± IQR)	70.0 ± 30 15.5 ± 4 1 ± 1 15.50 ± 6 3.0 ± 6 2.5 ± 6 22.5 ± 3.0 49.5 ± 6
***Objective assessment*** BMI (Kg/m^2^; median ± IQR) FEV_1_ (%; median ± IQR) VO_2_peak (mL/min; median ± IQR) predVO_2_max (%; median ± IQR) 6MWD (meters; median ± IQR) pred6MWD (%; median ± IQR) 1STST (repetitions; median ± IQR) pred1STST (%; median ± IQR) Glittre test (seconds; median ± IQR) predGlittre test (%; median ± IQR) Handgrip strength (Kgf; median ± IQR) predHandgrip strength (%; median ± IQR)	26.5 ± 3.3 54.0 ± 26 1,233 ± 792 61.5 ± 28.9 450.0 ± 124 87.1 ± 19.5 19.0 ± 5 61.5 ± 22.4 143.5 ± 54 80.4 ± 25.7 16.5 ± 8.0 55.1 ± 27.4

*IQR, Interquartile Range; EuroQoL, European quality-of-life scale; VAS, visual analog scale; CAT, Chronic obstructive pulmonary disease Assessment Test; mMRC, modified Medical Research Council scale; LCADL, London Chest activity of daily living scale; HADS, Hospital Anxiety and Depression Scale; A, anxiety; D, depression; MoCA, Montreal cognitive Assessment test; PRAISE, Pulmonary Rehabilitation Adapted Index of Self-Efficacy; BMI, Body Mass Index; FEV_1_, Forced Expiratory Volume in the first second; VO_2_peak, peak oxygen uptake; predVO_2_max, predicted maximum oxygen uptake; 6MWD, 6-min walk distance; pred6MWD, predicted 6MWD; 1STST, 1-min sit-to-stand test; pred1STST, predicted 1STST; predGlittre test, predicted Glittre test; predHandgrip strength, predicted handgrip strength*.

**Figure 2:**
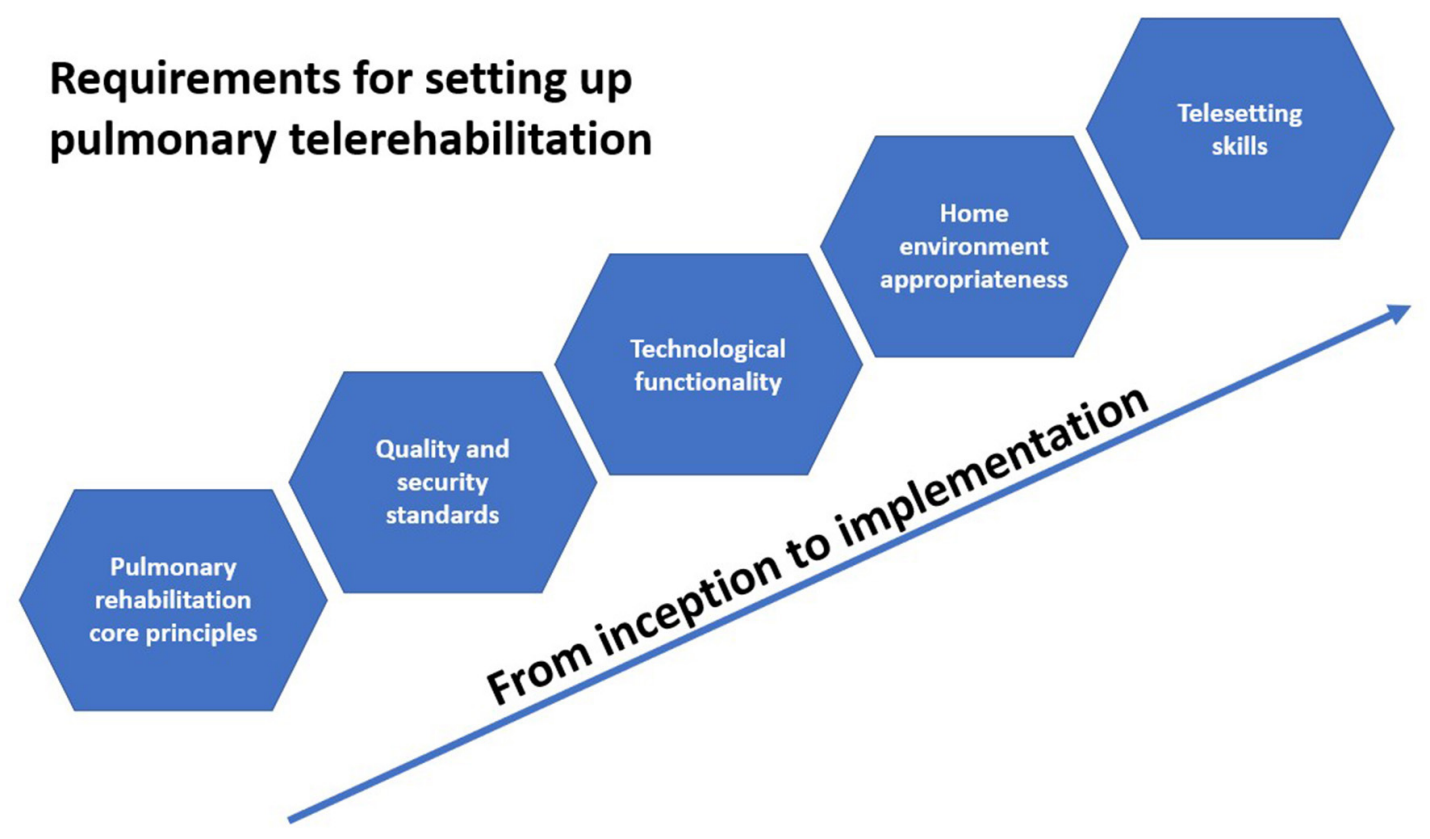
Requirements for setting up pulmonary telerehabilitation.

The inception of this pulmonary rehabilitation model integrated telerehabilitation as a continuum of care after a hospital setting period of 1 month. This configuration was important to achieve crucial requirements for telerehabilitation implementation. According to a consolidated framework for advancing implementation science, there are five major domains that influence implementation effectiveness: the intervention, inner and outer settings, the individuals involved, and the process by which implementation is accomplished (44). As a result of stakeholders' participatory engagement in pulmonary telerehabilitation implementation, this study proposes five major groups of requirements for setting up pulmonary telerehabilitation, as presented in Figure 2: pulmonary rehabilitation (PR) core principles, quality and security standards, technological functionality, home environment appropriateness, and telesetting skills. If the first two groups comprise pulmonary rehabilitation classical requirements regardless of implementation setting, the last three groups are telerehabilitation specific and a tacit knowledge to be considered.

Core pulmonary rehabilitation principle requirements were developed through strategies such as the following: (1) in-person individual exercise response profile evaluation; (2) in-loco patient warm-up, exercise, and cool-down learning phases; (3) presential contact with a pneumologist, physiotherapist, dietitian, and psychologist during self-management education sessions; (4) patient group intervention with individually engagement and commitment to the program by socializing among peers; and (5) a face-to-face therapeutic relationship built with the physiotherapist to later continue in the form of video-call telerehabilitation. This means that the face-to-face relationship with the physiotherapist was achieved throughout the first month with presential pulmonary rehabilitation sessions, including an objective assessment, subjective exam, and motivational interviewing techniques. In the second month of the program, when telerehabilitation was set, it was not required to establish a new relationship with a remote unfamiliar professional: communication was remote, but the physiotherapist was the same and the program dynamics continued without the need for a reset.

Quality and security standards were requirements important to be guaranteed prior to the onset of telerehabilitation and included strategies such as the following: (1) confirmation of patient address and phone contacts; (2) alternative contact plan with a caregiver, familiar, neighbor, or friend; (3) printed tutorial of tablet usage during telerehabilitation; and (4) individual action-plan as proposed by the Living Well with COPD™ program.

It was essential to ensure the technical functionality requirements were met on the week prior to the start of telerehabilitation, with a group intervention in the hospital setting helpful to (1) ensure first contact with tablet and earphones and a handling demonstration; (2) ensure first usage of domiciliary equipment to be delivered and in-person repetition of proceedings for self-monitoring; (3) enable simulation of a remote session with each patient acting solo while communicating via a tablet with the physiotherapist; and (4) focus attention on systematic self-monitoring with a registered routine as a method.

Ensuring home environment appropriateness requirements were met necessitated a dynamic strategy to succeed with the installation at each patient's home scenario. For this purpose, the physiotherapist teamed up with the logistics and transportation staff, and all equipment was delivered in-person to the patient with a domiciliary visit, adjusting the appropriateness of the setting to match the requirements of the telerehabilitation sessions.

Finally, requirements of telesetting skills were very important as add-ons in clinical practice. At the hospital, the physiotherapist had to be exclusively dedicated to telerehabilitation, and video calls were operationalized as sequences and were not continuous. This strategy was fundamental to achieving good quality video images and sound communications, optimizing time management and effectiveness with real-time remote monitoring and simultaneous registration of patient clinical files. Hence, the patient was scheduled for a first video contact with only the physiotherapist alone, and afterward, the rest of the team were video connected and disconnected dynamically, enrolling two different patients at the same time, as a strategy to preserve group identity and peer reinforcement. All patients were successful in providing feedback and accomplishing the purpose of exercise training. None of the patients had experienced previous health interventions by video call, and only one of the patients had previously used a tablet.

Table 3 presents pulmonary telerehabilitation outcomes. The difference between median values was statistically significant in CAT scores from 15.5 to 10.5 (*p* = 0.004), surpassing the minimal clinically important difference of 2 points. Also, there was a statistically significant difference between medians of PRAISE score from 49.5 to 53.0 (*p* = 0.006) close to the minimal clinically important difference of 3.59. The objective assessment excluded six participants due to imposed COVID-19 pandemic constraints for face-to-face hospital evaluations as a protocol.

**Table 3:** Pulmonary telerehabilitation outcomes.

	**Results at the end of the program**	***p*-value for median change different from zero**
**Subjective assessment (*****n*** **= 14)** EuroQoL—VAS (%; median ± IQR) CAT (score; median ± IQR) LCADL (score; median ± IQR) HADS A (score; median ± IQR) HADS D (score; median ± IQR) PRAISE (score; median ± IQR)	77.5 ± 50 10.5 ± 11 15 ± 4 2.5 ± 5 1.5 ± 4 53 ± 7	0.192 0.004^*^ 0.119 0.824 0.325 0.006^*^
**Objective assessment (*****n*** **= 8)** 6MWD (meters; median ± IQR) pred6MWD (%; median ± IQR) 1STST (repetitions; median ± IQR) pred1STST (%; median ± IQR) Glittre test (seconds; median ± IQR) predGlittre test (%; median ± IQR) Handgrip strength (Kgf; median ± IQR) predHandgrip strength (%; median ± IQR)	486.0 ± 77 88.2 ± 20.4 19 ± 11 69.0 ± 47.7 136 ± 56 76.6 ± 126.7 18 ± 7.6 59.2 ± 48.8	0.674 0.401 0.401 0.463 0.889 0.889 0.345 0.249

* *Statistically significant as p < 0.05 on related samples Wilcoxon Signed Rank test. IQR, Interquartile change; EuroQoL, European quality-of-life scale; VAS, visual analog scale; CAT, Chronic obstructive pulmonary disease Assessment Test; LCADL, London Chest activity of daily living scale; HADS, Hospital Anxiety and Depression Scale; A, anxiety; D, depression; PRAISE, Pulmonary Rehabilitation Adapted Index of Self-Efficacy; 6MWD, six-minute walk distance; pred6MWD, predicted 6MWD; 1STST, one-minute sit-to-stand test, pred1STST, predicted 1STST; predGlittre test, predicted Glittre test; predHandgrip strength, predicted handgrip strength*.

When the pulmonary rehabilitation program concluded, patients reported a mean level of satisfaction with rehabilitation goals achievement scores of 88.1 ± 8.6% (range between 80 and 100%). Also, patients reported a mean level of satisfaction values with pulmonary rehabilitation with telerehabilitation experienced as a model of care of 95.4 ± 6.3% (range between 80 and 100%).

## Discussion

The engagement of all stakeholders in the participatory research process, especially the patients, was a cornerstone for the success of telerehabilitation implementation. Such a methodological benefit has also been described in optimizing the implementation of pulmonary rehabilitation for COPD patients with limited accessibility due to geographical distances (45) and also in telerehabilitation redesigned for underserved Hispanic and African American patients with COPD (46).

It is important to acknowledge a core set of conceptual reasons for selecting telerehabilitation as an appropriate model of care to develop. As pulmonary rehabilitation is widely underutilized and frequently inaccessible to patients (47), current programs need to be upgraded on means of delivery to boost an effective waiting-list reduction. For this purpose, there has been a significant telemedicine contribution to modernizing healthcare within the framework of integrated care and the chronic care model of disease management (48). No less relevant is the fact that the provision of care needs to be redesigned to promote patient autonomy and self-efficacy (49), and telerehabilitation provides remote care to take place exactly where change needs to happen: in the patients' own living environments. All of these reasons prevail and became even more relevant, making telerehabilitation a new standard in a Post-COVID-19 world (50).

Altogether we have found five major groups of requirements for setting up pulmonary telerehabilitation: (1) pulmonary rehabilitation core principles, (2) quality and security standards, (3) technological functionality, (4) home environment appropriateness, and (5) telesetting skills. Such a framework led to the development of operational strategies aiming to empower patient self-efficacy with increased digital skills and health literacy, promote safety and quality conditions for real-time remote person-centered care, and optimize the efficiency of the exercise-based intervention with tech-enabled remote healthcare. We believe that such operational experience is an advantageous background to consider for those redesigning upgraded telerehabilitation programs.

A noteworthy remark is that the onset of this telerehabilitation model was supported by hospital services and human resources and required additional costs to the standard of care, including logistics, transport, and communications, as briefly described. This proof-of-concept research demonstrated the feasibility of telerehabilitation implementation in a setting before the COVID-19 pandemic. A further step was to prove beyond the clinical benefit a cost-benefit analysis of pulmonary rehabilitation standard-of-care vs. pulmonary telerehabilitation innovation by means of an economic study, which was beyond the scope of the present study. Interesting is that what used to be a struggle for investment, mainly regarding costly communication expenses, is nowadays a widespread area of funding with promising exponential development over the years to come.

This study describes an increase in patients' reported quality of life and perceived self-efficacy with telerehabilitation, a finding that requires future research with a larger sample to strengthen the evidence. An important outcome to emphasize was the high level of patient satisfaction not only about rehabilitation goals achievement but more importantly about pulmonary rehabilitation with telerehabilitation experienced. Such patient-reported outcomes and experiences support telerehabilitation as an emerging model of care with high acceptance from its end-users. With the COVID-19 pandemic, this was one of the many ongoing trials that suspended some or all research activities (51), as it was no exception to global rehabilitation services that experienced partial or complete disruption (52). Because of this, a major study limitation is the incomplete objective patient outcomes assessment, as face-to-face hospital evaluations as a protocol were no longer authorized due to the declared COVID-19 pandemic. Also, external validity may be limited, as there might be various frameworks within telerehabilitation developers, given possible multiple operational scenarios with different requirements for telerehabilitation setting up and implementation. This includes digital health literacy levels of both patients and healthcare professionals (53). Therefore, future research collecting global multicentric experiences of telerehabilitation implementation might be relevant to leverage an upgraded restart of the pulmonary rehabilitation study outside of the pandemic scenario.

Current worldwide developments have made pulmonary telerehabilitation rise rapidly from being a promising area of research (49, 54, 55) to a required shift to maintain ongoing pulmonary rehabilitation programs during the COVID-19 pandemic (52, 56, 57). This makes the reported operational experience resourceful to those who are engaged with telerehabilitation in the frontline, not as an add-on but instead as a necessary intervention. Naturally, emerging models will continue to evolve and produce increased diversity in pulmonary rehabilitation delivery of care (4), as nowadays telerehabilitation plays a major part in the solution to improve the effectiveness, accessibility, and resilience of healthcare systems worldwide.

## Data Availability Statement

The original contributions presented in the study are included in the article/supplementary material, further inquiries can be directed to the corresponding author/s.

## Ethics Statement

The studies involving human participants were reviewed and approved by Ethics Committee of Centro Hospitalar Universitário Lisboa Norte, and Centro Académico de Medicina de Lisboa (number 43/17). The patients/participants provided their written informed consent to participate in this study.

## Author Contributions

CS: conceptualization, methodology, investigation, formal analysis, and writing–original draft. FR: conceptualization, investigation, and writing—review and editing. CC: writing—review and editing. CB: conceptualization and writing—review and editing. All authors edited the manuscript and approved the final draft.

## Funding

This work was supported by Fundação para a Ciência e Tecnologia (FCT) and Nippon Gases Portugal under the Ph.D. studentship in Industry grant PDE/BDE/127785/2016 awarded to CS.

## Conflict of Interest

CC is employed by Nippon Gases Portugal. The remaining authors declare that the research was conducted in the absence of any commercial or financial relationships that could be construed as a potential conflict of interest.

## Publisher's Note

All claims expressed in this article are solely those of the authors and do not necessarily represent those of their affiliated organizations, or those of the publisher, the editors and the reviewers. Any product that may be evaluated in this article, or claim that may be made by its manufacturer, is not guaranteed or endorsed by the publisher.
